# Vertical transmission and seasonal dimorphism of eriophyoid mites (Acariformes, Eriophyoidea) parasitic on the Norway maple: a case study

**DOI:** 10.1098/rsos.220820

**Published:** 2022-09-21

**Authors:** Philipp E. Chetverikov, Pavel B. Klimov, Qixin He

**Affiliations:** ^1^ Russian Academy of Sciences, Zoological Institute, Universitetskaya nab. 1, 199034, St. Petersburg, Russia; ^2^ X-BIO institute, Tyumen State University, 6 Volodarskogo Str., Tyumen 625003, Russia; ^3^ Purdue University, West Lafayette, IN, USA

**Keywords:** deuterogyny, dispersal, host specificity, seasonality

## Abstract

Eriophyoid mites are highly host-specific, microscopic phytoparasites that primarily disperse to new hosts passively via wind. This seems paradoxical, as the likelihood of landing on an appropriate host species needed to survive appears low. Here we investigate two eriophyoids found on the Norway maple *Acer platanoides*: *Aceria platanoidea* and *Shevtchenkella serrata*. For 14 months, we observed mite phenotypical changes and micro-habitat distribution on host plants and their propagules. Both mite species hibernate on twigs or samaras fallen on the ground, and, in the spring, feed on buds or seedlings, respectively. This apparently novel association with plant seeds indicates that the mites can exploit the host dispersal mechanism and colonize the next generation of hosts (vertical transmission). Our seasonal and DNA sequence data also indicate that *S. serrata* has two distinct morphotypes that partially overlap seasonally. This work can provide new insights into the dispersal routes of eriophyoid mites and transmission patterns of plant pathogens vectored by these mites, with implications for better pest mite species control.

## 1. Introduction

**Figure 1.**
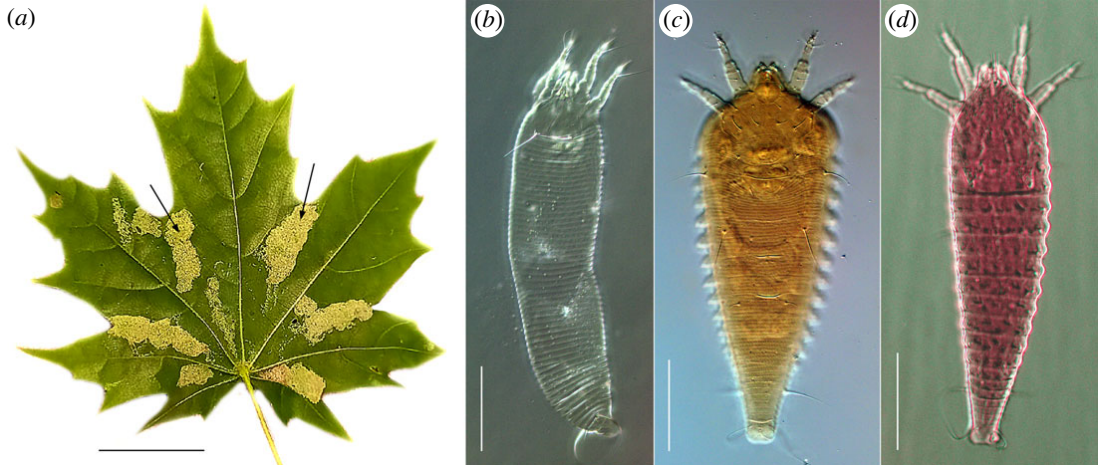
Eriophyoid mites are morphologically simplified, microscopic chelicerates that are capable of inducing a range of gall formations on plants. (*a*)—leaf erinea (arrows) induced by *Aceria platanoidea* on the Norway maple, (*b*)—dorsal view of the female of *A. platanoidea* (light microscopy), (*c*)—dorsal view of the summer (protogyne) female of *Shevtchenkella serrata*, (*d*)—dorsal view of the winter (deutogyne) female of *S. serrata*. Scale bars: (*a*) = 3 cm; (*b*) = 1 cm; (*c*), (*d*) = 50 µm.

Eriophyoidea is an extremely diverse superfamily of mites that feed on plants, with each species generally specializing in only one or a few closely related host genera [1]. About 20% of species induce various galls [2], including felted patches of hairs called erinea (figure 1). Most eriophyoids cause little damage to their hosts; at low numbers, they may even benefit the host plant by serving as an alternate food source for natural predators (i.e. phytoseiid mites) of dangerous pests (i.e. tetranychid mites) [3–5]. Several species are economically important pests transmitting viruses and damaging agricultural and ornamental crops [6–8] or are beneficial for biocontrol of weeds [8–10]. Being parasitic, eriophyoids have undergone adaptive morphological simplification, having only four legs, few setae, no true eyes and a worm-like body [11–13]. Averaging approximately 200–300 µm, these mites are nearly invisible to the unaided eye. Detailed behavioural studies of eriophyoids are limited [3,14].

Most eriophyoids have only one female morphotype in their life cycle, called the protogyne or ‘summer female’; and only approximately 150 of approximately 4400 currently described species have an additional cold-tolerant female morphotype called deutogyne [15–17]. Morphologically, deutogynes can appear nearly indistinguishable from protogynes [17–19] or be very divergent. Some of these deutogynes have been placed in different genera or subfamilies—misclassifications later detected by molecular techniques [20].

Eriophyoid usually disperse via air currents, and less frequently by rain, snow or phoresy [14,21,22]. Except for the latter, these dispersal strategies depend on random effects and may fail if the host density is low and dispersing mite infrapopulation size is small. Dispersal via vertical transmission may ensure better success in colonization of new host individuals [23,24], but this aspect is poorly known in eriophyoid mites.

Here, we provide seasonal observations of eriophyoids associated with a single, heavily infested population of Norway maples (*Acer platanoides* L.). This tree is the most widespread fast-growing native maple in Europe and is invasive in eastern North America [25]. Seeds are disseminated by wind via specialized winged fruits called samaras, scattering up to 25 m from the parental tree depending on the wind power [26]. We provide observational evidence pointing to vertical transmission of eriophyoid mites via samaras, as well as reporting previously undescribed seasonal dimorphism.

## 2. Material and methods

### 2.1. Field observations

From July 2020 to October 2021 (excluding mid-December to March), we sampled eriophyoids from native *Acer platanoides* (Sapindaceae) in an approximately 60 m × 60 m mixed evergreen-deciduous forest in Vyritza, Leningrad Oblast, Russia. We sampled five trees every two weeks and 10 trees every one to two months; all these trees were mature (20–40 years old). Every time we sampled three twigs from a tree, each twig with 3–8 buds/leaves and 3–8 samaras (if present). Additionally, we sampled plant material around the target trees (fallen maple leaves and samaras) and young plants—seedlings (1–100 days old) and saplings (2–5 years old). We separately bagged vegetative parts as well as propagules and examined them in the lab. Over 14 months, for trees 1–5, we recorded mite abundance (as irregular intervals) from 645 leaves, 488 buds, 72 inflorescences, 276 samaras attached to the tree, 124 samaras and 57 leaves on ground, 82 seedlings and 28 saplings. Additional presence/absence data were collected for trees 6–15. We also surveyed samaras and seedlings (*n* = 36) under the maple trees and outside this area and recorded the exact mite count data.

### 2.2. Abundance data

Field observations data were converted into a dataset recording date, day and night temperature, sampled plant parts (*leaves*, *samaras*, *buds*, *leaves*_*fallen*, *samaras*_*fallen*, *seedlings*, *flowers*, *saplings*), and mite species (AP – *Aceria platanoidea*, SS – *Shevtchenkella serrata*) and morphotypes (p – protogyne, d – deutogyne). Our main dataset had interval and count data (mostly trees 1–5); while our confirmatory dataset had presence/absence data only (trees 6–15). For each plant category, mite abundance data were re-coded to reduce the number of the original intervals. The main abundance dataset was visualized using a custom R script. Raw data, abundance dataset, scripts and analysis detail are available here [27].

### 2.3. Statistical tests

To test whether the number of mites on samaras or seedlings is different in areas under a maple tree (A1) and outside, 20 m from the source in an area lacking maple trees (A2), we used the Fisher's exact test implemented in R. H0 = mite communities (species composition and richness) among the two areas are not statistically different. This would indicate that mites cannot be transmitted by wind as wind dispersal is distance-dependent. H1 = under the wind-dispersal scenario, mite communities should be statistically different, particularly mites should be more abundant in A1 and less abundant in A2. Because wind dispersal is passive, we consider each wind transmission event as independent, i.e., wind transmission is not affected by a host tree or mite genetic background. In case there are dispersal-related differences in mites and there is a bias in the distribution of these differences in our local population, this test may not be representative of the entire population. Raw data, scripts and analysis detail are available here [27].

### 2.4. Collection and morphological analysis

Eriophyoids were removed using a dissecting microscope and a fine minuten pin; individuals of a single morphotype were placed in Eppendorf tubes filled with 96% ethanol. Mites were also mounted in a modified Berlese's medium with iodine, cleared [28], and examined under a compound microscope equipped with differential interference contrast optics (DIC).

### 2.5. DNA extraction and sequencing

One to three mite specimens of each morphotype were used per reaction to amplify the fragments of two genes via PCR: mitochondrial cytochrome oxidase c subunit I gene (Cox1) and a nearly complete fragment of nuclear 28S ribosomal DNA (rDNA). DNA extraction, thermocycling and sequencing profiles, procedures and primer sequences follow [29]. Sequences were deposited in GenBank and Kimura-2-parameter (K2P) distances were determined with Mega X [30,31].

## 3. Results

### 3.1. Field observations

**Figure 2.**
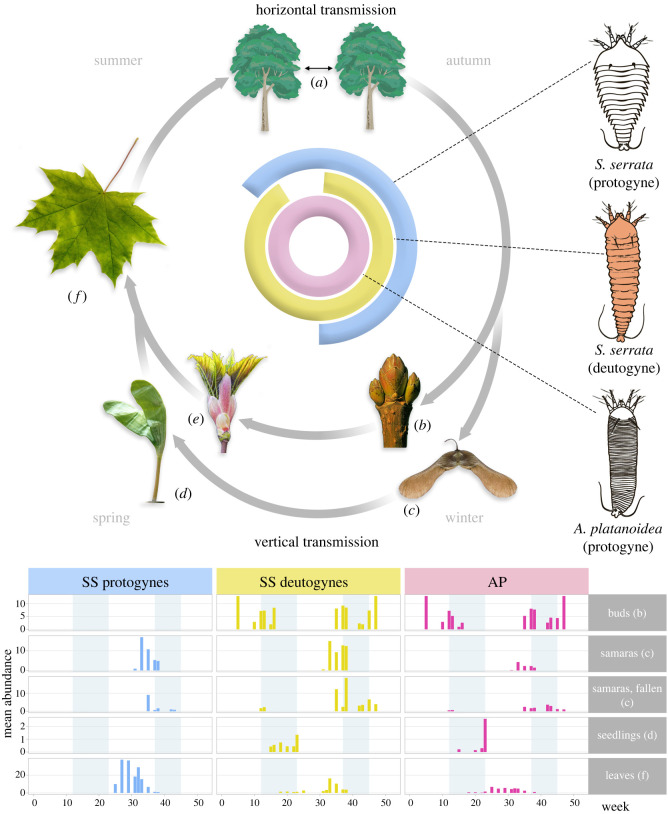
Seasonal distribution of *Shevtchenkella serrata* protogynes (p*SS*), deutogynes (d*SS*), and *Aceria platanoidea* (*AP*) protogynes (p*AP*) on the Norway maple. During the plant growing season, mites are primarily horizontally transmitted via wind (*a*). All three mite groups overwinter near buds on twigs (*b*) or on fallen samaras (*c*). After frost, only d*SS* and p*AP* were found on seedlings (*d*), buds (*e*) and expanded leaves (*f*). *AP* induces erineum galls on leaves (not shown), galls primarily occupied by p*AP*, with d*SS* occasionally found as inquilines. In mid-June, the first p*SS* appeared and d*SS* diminished, with d*SS* absent in July. From August until leaf fall, all three female groups (p*SS*, p*SS*, p*AP*) were detected on leaves and fruits, then again on fallen samaras and near buds. No sampling was done from late December to March when samaras were embedded in frozen leaf litter. For simplicity, on the circular plot (top), time intervals are approximate and not to scale. Time intervals are exact on the mite abundance histograms (bottom); the four seasons are shown by alternate shading.

In August 2020, protogynes and deutogynes of *Shevtchenkella serrata* (Nalepa, 1892) [32] (abbreviated p*SS* and d*SS* below), and protogyne females of *Aceria platanoidea* (Nalepa, 1922) [33] (abbreviated p*AP* below), were all found feeding on abaxial leaf surfaces, either as vagrants (*SS*) or as gall-formers (*AP* in erineum), as well as on immature fruits still attached to the parent trees. From autumn to early December, we observed mixed groups consisting of 5–20 motionless p*SS*, d*SS* and p*AP* hibernating near terminal buds, on stems, and in crevices on fallen samaras (figure 2).

In autumn 2020 and spring 2021, no mites were found on approximately 60 leaves on the ground. We did not sample from late December 2020 to March 2021 when samaras were embedded in frozen leaf litter. Motionless d*SS* were detected after the snow had completely melted in late March 2021. Eight motionless d*SS* and three p*AP* were found on 21 March 2021 on three fallen samaras on the ground in an area lacking decomposing maple leaves. Light microscopy of partially cleared mites indicated that four d*SS* and two p*AP* were alive (all contained approximately 20 sperm cells within the spermatheca and developing eggs), while five other mites were dead. At this time, groups of motionless, hibernating d*SS* and p*AP* females were found near the buds on twigs of the target trees probably still in diapause and therefore not capable of dispersal by wind as they are attached to plant periderm with their anal suckers and secretions of the anal glands, and cannot acquire the characteristic wind-dispersal posture (standing straight with their legs up) [18]. Our statistical tests show that the mite communities (species composition and abundance) found on fallen samaras or seedlings under the host tree and in maple-free areas (20 m away from the source) were not statistically different (Fisher's exact test, *p* > 0.05), supporting the null hypothesis (no wind dispersal from mature trees to propagules on the ground). This indicates that the presence of mites on fallen samaras or seedlings is likely due to vertical transmission. The first maple seedlings appeared in the beginning of April, two weeks after the last night frost. Since that time, we found d*SS* on seedlings, e.g. 1–2 d*SS* were found on hypocotyls and cotyledons of 12 of the 32 seedlings on April 18th. Because in early April some mites were found on seedlings from samaras found outside areas covered by old maple leaves, wind transmission of these mite individuals is unlikely.

One month later, d*SS* and p*AP* were found on cotyledons, petiole bases and abaxial leaf surfaces of seedlings, saplings, and mature maple trees. Erinea were first observed on May 29th, with 1–2 *AP* females and eggs found between abnormal trichomes on the abaxial surface of true leaves of seedlings, saplings and mature trees; 4–5 d*SS* were on abaxial leaf surfaces, some as inquilines within *AP-*induced erinea.

In mid-June 2021, numerous p*SS* and immatures of *SS* appeared on the same mature tree branches sampled in spring 2021 and on seedlings and saplings along with p*AP* (in erinea), whereas d*SS* were few. During July 2021, there were no d*SS*, while hundreds of p*SS* were present on most leaves; erinea with dense *AP* populations (including numerous immatures and a few males which appeared only in July) were also common. In August 2021, we again detected the two *SS* female morphs (d*SS*, p*SS*) and p*AP* on leaves; and from mid-August to the beginning of leaf abscission in the fall, d*SS*, p*SS* and p*AP* were present on leaves and fruits. Later, we again detected these three female groups on samaras on the ground.

### 3.2. Molecular analysis of seasonal morphotypes

Based on morphological traits alone, deutogynes of *SS* could be classified as members of the genus *Anthocoptes* (a full taxonomic description will be presented elsewhere). However, their identity as *SS* was initially suspected when an aberration in the seasonal data was observed, and was confirmed by DNA sequence data [34]. Cox1 sequences of deutogynes (*n* = 5, GenBank accession OK489799) and protogynes (*n* = 6, OK489800) were identical, while 28S rDNA sequences (OK491613 and OK491614, respectively) were nearly identical (K2P distance = 0.001%). In comparison to protogynes, deutogynes are orange rather than white and have a narrower body without lateral projections (figure 1*c,d*). 28S rDNA of p*AP* from early spring versus midsummer was also sequenced (OK491612 and OK569898, respectively; K2P distance = 0.000%).

## 4. Discussion

### 4.1. Seasonal dimorphism

While the morphology of *AP* was constant throughout the seasons, *SS* had a pronounced female dimorphism, with two morphotypes exhibiting partially overlapping emergence times. One SS morphotype (deutogyne) was absent in the midsummer and another (protogyne) was absent from winter to early summer (figure 2). We confirmed the identity of the two morphs by DNA sequencing. In the past, resolutions of taxonomic confusion associated with dimorphic females required laborious and time-consuming observations of mite life cycles [15,18], however, nowadays, these issues can be resolved by DNA sequencing [17,20].

### 4.2. Vertical transmission

Most eriophyoids live on foliage and are wind dispersed—a risky, and primarily horizontal, form of transmission [14,16,35]. However, our data and a few published reports indicate that a portion of eriophyoid population from the same plant host may also live on the plant's generative organs and fruits [36]. These mite individuals, therefore, may disperse directly from host parents to offspring, thus reducing the risk of stochastic events associated with wind dispersal. Our data indicate that in the two eriophyoid mite species, vertical transmission may be a distinct dispersal strategy along with the regular transmission route by the wind. We suggest that vertical transmission is an important but currently overlooked eriophyoid dispersal mechanism and could be a factor in the development of host specificity and high diversity of these mites. Pathogens transmitted both vertically and horizontally are more likely to be less virulent than those transmitted horizontally alone, as dispersal is dependent on host reproductive success [37]. Additionally, the transition from horizontal to vertical transmission of a symbiont has been linked to the evolution from a parasitic to a mutualist lifestyle [24]. It was also shown that the wheat curl mite may contain two separate ‘dispersal castes’ differing in transmission behaviour [35]. Additional research, further quantitative data and statistical analyses are needed to determine whether in our system (a) the observed variation in hibernation sites is a random and density-dependent effect or (b) mite aggregations on the plant generative organs are non-random and independent of the mite infrapopulation density. In the latter case, it would be interesting to estimate the rate of horizontal versus vertical transmission and compare with the rate of successful colonization for both types of transmission.

## 5. Conclusion

Here we report parasites synchronizing their ecology and life cycles to optimally exploit seasonally available host resources. In this system, horizontal mite dispersal by wind may be a risky strategy, especially if the host abundance is low and/or mite infrapopulation size is small. However, a portion of mites may migrate on plant fruits and colonize new host individuals vertically. To advance our knowledge on how vertical transmission, deuterogyny, and microhabitat migration can contribute to the coevolution of eriophyoids and their plant hosts, further experimental data and analyses are needed. Our work suggests that well-timed removal of plant material fallen on the ground or applying mulch are important for preventing uncontrolled germination of potentially mite-infested seeds. Finally, broader application of pesticides (including biocontrol agents, e.g. predatory mites) at the soil level may contribute to control vertically transmitted pests on seedlings.

## Data Availability

DNA sequences: GenBank accessions OK489799, OK489800, OK491612, OK491613, OK491614, OK569898 [34]. The data are provided in electronic supplementary material [27].
